# Selection and Characterization of Palmitic Acid Responsive Patients with an OXPHOS Complex I Defect

**DOI:** 10.3389/fnmol.2017.00336

**Published:** 2017-10-18

**Authors:** Tom E. J. Theunissen, Mike Gerards, Debby M. E. I. Hellebrekers, Florence H. van Tienen, Rick Kamps, Suzanne C. E. H. Sallevelt, Elvira N. M. M.-D. Hartog, Hans R. Scholte, Robert M. Verdijk, Kees Schoonderwoerd, Irenaeus F. M. de Coo, Radek Szklarczyk, Hubert J. M. Smeets

**Affiliations:** ^1^Department of Clinical Genetics, Maastricht University Medical Centre, Maastricht, Netherlands; ^2^Department of Genetics and Cell Biology, School for Oncology and Developmental Biology, Maastricht University Medical Centre, Maastricht, Netherlands; ^3^Maastricht Centre for Systems Biology, Maastricht University, Maastricht, Netherlands; ^4^Department of Neurology, Erasmus Medical Center, Rotterdam, Netherlands; ^5^Department of Clinical Genetics, Erasmus Medical Center, Rotterdam, Netherlands; ^6^Department of Pathology, Erasmus Medical Center, Rotterdam, Netherlands

**Keywords:** high-fat diet, palmitic acid, complex I deficiency, TMEM126B, assembly factors

## Abstract

Mitochondrial disorders are genetically and clinically heterogeneous, mainly affecting high energy-demanding organs due to impaired oxidative phosphorylation (OXPHOS). Currently, effective treatments for OXPHOS defects, with complex I deficiency being the most prevalent, are not available. Yet, clinical practice has shown that some complex I deficient patients benefit from a high-fat or ketogenic diet, but it is unclear how these therapeutic diets influence mitochondrial function and more importantly, which complex I patients could benefit from such treatment. Dietary studies in a complex I deficient patient with exercise intolerance showed increased muscle endurance on a high-fat diet compared to a high-carbohydrate diet. We performed whole-exome sequencing to characterize the genetic defect. A pathogenic homozygous p.G212V missense mutation was identified in the *TMEM126B* gene, encoding an early assembly factor of complex I. A complementation study in fibroblasts confirmed that the p.G212V mutation caused the complex I deficiency. The mechanism turned out to be an incomplete assembly of the peripheral arm of complex I, leading to a decrease in the amount of mature complex I. The patient clinically improved on a high-fat diet, which was supported by the 25% increase in maximal OXPHOS capacity in TMEM126B defective fibroblast by the saturated fatty acid palmitic acid, whereas oleic acid did not have any effect in those fibroblasts. Fibroblasts of other patients with a characterized complex I gene defect were tested in the same way. Patient fibroblasts with complex I defects in NDUFS7 and NDUFAF5 responded to palmitic acid, whereas ACAD9, NDUFA12, and NDUFV2 defects were non-responding. Although the data are too limited to draw a definite conclusion on the mechanism, there is a tendency that protein defects involved in early assembly complexes, improve with palmitic acid, whereas proteins defects involved in late assembly, do not. Our data show at a clinical and biochemical level that a high fat diet can be beneficial for complex I patients and that our cell line assay will be an easy tool for the selection of patients, who might potentially benefit from this therapeutic diet.

## Introduction

Mitochondrial disorders are genetically and clinically heterogeneous metabolic disorders with a prevalence of ∼1 in 5,000 in the general population (Skladal et al., 2003; Gorman et al., 2015). Especially high energy-demanding organs are affected as a result of impaired oxidative phosphorylation (OXPHOS), commonly manifesting with pronounced neuromuscular symptoms, fatigability and exercise intolerance. The subunits of the OXPHOS machinery are encoded by both the nuclear and mitochondrial DNA and pathogenic mutations in either genome can affect OXPHOS capacity, often presenting with isolated or combined mitochondrial complex deficiencies. Complex I deficiency (NADH: ubiquinone oxidoreductase) is the most prevalent OXPHOS disorder and is found either in isolation or combined with deficiencies of the other complexes (McFarland et al., 2004). Treatment options in complex I deficiencies are currently limited, despite extensive research on a variety of approaches, and successes are often anecdotic and not applicable to all patients. Clinical improvements as a result of high-fat intake with or without carbohydrate restriction have been reported for some of the complex I deficient patients and have led to the prescription of high-fat and ketogenic diets to patients with complex I, but also with pyruvate dehydrogenase complex deficiencies, Leigh syndrome, epilepsy and Parkinson disease (Wijburg et al., 1992; Roef et al., 2002; Kang et al., 2007; Baranano and Hartman, 2008; Rogovik and Goldman, 2010; Paleologou et al., 2017; Sofou et al., 2017). The resulting increase in plasma fatty acids and deprivation of carbohydrates is expected to cause a shift from a glycolytic energy metabolism to the beta-oxidation of fatty acids, which is accompanied by the production of ketone bodies in the liver mitochondria (Peters and Leblanc, 2004; Stanley et al., 2014; Paoli et al., 2015). Ketone bodies (3-beta-hydroxybutyrate, acetoacetate, and acetone) are short-chain organic acids that can freely diffuse across cell membranes and were suggested to serve as an alternative energy substrate to glucose for the brain (Hasselbalch et al., 1995; Scholl-Burgi et al., 2015). The ketogenic diet was shown to have beneficial effects for patients with pyruvate dehydrogenase complex deficiency related brain pathology, especially in case of pharmaco-resistant epilepsy (Sofou et al., 2017). Although different mechanisms of action, involving both ketone bodies and fatty acids, have been proposed (Schwartzkroin, 1999; Fraser et al., 2003; Bough et al., 2006; Kim and Rho, 2008; Xu et al., 2008; Maalouf et al., 2009; Jeong et al., 2011), the precise mechanisms are not clear. Also for the treatment of mitochondrial myopathies due to complex I deficiency the therapeutic use of high-fat diets has been recommended, as supplementation of fatty acids via direct triacylglycerol infusion was shown to significantly improve exercise endurance in some patients (Roef et al., 2002). The latter observation was explained by an increase in mitochondrial beta-oxidation, which results in relatively higher FADH_2_:NADH^+^+H^+^ ratios than the carbohydrate driven tricarboxylic acid cycle, thereby enabling bypassing of the complex I defect (Roef et al., 2002; Paoli et al., 2014; Steriade et al., 2014). Yet, this mechanism cannot by itself explain why only some of the complex I deficient patients respond to high-fat treatment, suggesting that these treatments influence the OXPHOS system, at least in part, in a gene- or protein-specific manner (Hughes et al., 2014; Frey et al., 2017).

Despite the clinical application of high-fat diets, it is yet unknown which genetic complex I deficiencies could benefit from these diets. In this paper, we characterize the gene defect in a patient with complex I deficiency using whole-exome sequencing, who clinically improved on a high-fat diet. We tested the *in vitro* responsiveness to the saturated fatty acid palmitic acid of this patient and other patients with a genetically characterized complex I deficiency. This simple *in vitro* assay could be used in a clinical setting to select the patients who could benefit from this treatment.

## Materials and Methods

### Patient

A Dutch female patient, who has a healthy younger brother and gave birth to a healthy child, was subject to HPLC based metabolite profiling on blood-plasma and urine to reveal metabolic abnormalities. A Quadriceps muscle biopsy was taken to spectrophotometrically measure OXPHOS complex activities for complex I, II+III, IV, and V, normalized to citrate synthase activity, according to a method described by Sgobbo et al. (2007). Muscle histochemistry was performed by staining the succinate dehydrogenase (SDH) and cytochrome oxidase (COX) complexes and electron microscopy was performed to identify abnormalities in mitochondrial morphology and quantity. A skin biopsy was taken from the TMEM126B patient and dermal fibroblast were isolated in a similar manner (as described below) as previously executed for the five other patients with complex I deficiency (NDUFS7, NDUFAF5, ACAD9, NDUFA12, NDUFV2 defective fibroblasts), who were all biochemically and genetically diagnosed at the Maastricht University Medical Centre (MUMC+).

### Dermal Fibroblast Sampling, Isolation and Culturing

Primary dermal fibroblasts from the patient were obtained by taking a 3–5 mm punch biopsy from the arm, which was cut into 18 smaller pieces and 3 biopsy pieces were transferred to each gelatin pre-coated 6-well containing Dulbecco’s Modified Eagle Medium (DMEM, 25 mM glucose) supplemented with 20% fetal bovine serum (FBS). When reaching confluency, fibroblasts were trypsinized and transferred to a T75 flask, and, after 3 passages, immuno-stained to check HSP-47 positivity. During later passages fibroblasts were cultured in DMEM (25 mM glucose) supplemented with 10% FBS and 0.2% Penicillin-Streptomycin.

### Dietary Studies

The patient’s (TMEM126B patient) muscle endurance was examined by performing a bicycle test at 15% of the maximal capacity (15% of *W*_max_), based on the patient’s usual diet in which 34,5% of the energy was derived from fat and 49,4% from carbo-hydrates. Magnetic Resonance Phosphor Imaging (31P MRS) of the muscle was performed to measure muscle recovery. A dietary intervention study was performed, where the patient was first prescribed to consume a high-carbohydrate diet for 3 weeks, where 25% of the energy derived from fat, and subsequently a high fat-diet, where 55% of the energy was derived from fat intake, to study the effects of dietary intake on muscle endurance, measured by the bicycle test at 15% of *W*_max_. Additionally, differences in muscle endurance and strength were examined with direct substrate infusion, comparing intra-lipid infusion (3,7 mg/kg/min) to glucose infusion (10 mg/kg/min), and measuring muscle strength at different body parts with intervals of relaxation.

### Whole-Exome Sequencing

Blood DNA was fragmented and exons were captured using the Agilent SureSelect version 4, exome enrichment kit, including UTRs (Agilent Technologies, Santa Clara, CA, United States). Sequencing was performed on an Illumina HiSeq2000 platform, using a 2 × 100 bp paired end setting (Illumina, San Diego, CA, United States). Bcl2fastq 1.8.4 allowed base-calling and demultiplexing; BWA 0.5.9 was used for read alignment against human reference genome hg19. Duplicate reads were removed by Picard software suite 1.77 (Broad Institute); variant calling was performed with GATK 2.1-8 (Broad Institute). An in-house data analysis pipeline extracted database information on annotated variants according to the UCSC RefGene track; dbSNP137 and dbNSFP v2.0. Exome data were filtered for homozygous and heterozygous variants with allele frequencies lower than 1% (dbSNP137) and a coverage of >10 reads, consisting of non-synonymous substitutions, INDELs (in-frame and frameshift), nonsense mutations and splice-variants. Non-annotated variants were maintained, unless allele frequencies exceeded 5% in our in-house patient database. Missense variants in moderately to highly conserved domains were maintained. Pathogenicity of non-synonymous missense mutation was estimated by Polymorphism Phenotyping-2 (PolyPhen-2), Sorting Intolerant From Tolerant (SIFT) and PROVEAN.

### Sanger Sequencing and QPCR

Mutation segregation analysis was performed on blood DNA from the healthy mother and index patient. The father was not available for further analysis. Primers were designed to cover the exon 5 missense mutation in *TMEM126B* (transmembrane protein 126B, NC_000011.10) (Supplementary Table S2). The *TMEM126B* nDNA copy number was quantified by the 7900HT Fast Real-Time PCR System, normalizing to nuclear *B2M* (nuclear gene beta-2-microglobulin, NC_000015.10). Primers were designed in exons 1, 2, and 5 (Supplementary Table S3) and amplification was performed using Sensimix Sybr Hi-Rox (Bioline, Taunton, MA, United States).

### Blue Native-Polyacrylamide Gel Electrophoresis

Mitoplasts were isolated from fibroblasts of the index patient, control 1 (C1) fibroblasts, which were Normal Human Dermal Fibroblasts (NHDF), control 2 (C2) fibroblasts derived from a single healthy individual, and an *NDUFA9* mutant as previously described (Nijtmans et al., 2002). 25 μg mitoplast fraction was loaded on a 4–16% polyacrylamide gradient gel (Invitrogen), after quantification by Qubit Protein Assay Kit (Thermo Fisher Scientific, Waltham, MA, United States). Western blot analysis was performed by hybridization with monoclonal antibodies for complex I subunit NDUFA5, NDUFB8 and complex II (SDHA) as a protein loading control (Mitosciences, Eugene, OR, United States).

### Cloning and Lentiviral Complementation

*TMEM126B* cDNA (isoform A, NM_018480.4) was cloned into a 3rd generation pUltra-Chili lentiviral backbone vector, containing a tomato-red reporter gene for validating viral transduction efficiency (Addgene, Malcolm Moore Lab). The p.G212V mutation was introduced by side directed mutagenesis according to the QuikChange II Site-Directed Mutagenesis Kit (Agilent Technologies). Transfection of HEK293FT viral producing cells was accomplished by combining transIT (Mirus Bio), 3rd generation packaging and envelope plasmids (Rev, Gag and Pol, VSV-G) and the backbone expression vector. 48 h post transfection, media containing viral particles were collected and filtered (0.45 μm), subsequently polybrene was added (8ug/ml). Patient fibroblast were seeded at 30% confluency and transduced for 24 h, resulting in a transduction efficiency of >90%.

### Seahorse Analysis

Patient (*TMEM126B*) fibroblasts, control 1 (C1) fibroblasts, which were Normal Human Dermal Fibroblasts (NHDF), and control 2 (C2) fibroblasts, deriving from a single healthy individual, were seeded at 22,000 cells per well, using 12 wells per condition, and attached overnight in standard DMEM (25 mM glucose) supplemented with FBS (10%). One hour prior to assay, media was replaced with Seahorse assay media containing 10 mM glucose, 2 mM glutamine, 1 mM pyruvate, 10% FBS, and oxygen consumption rates (OCR) were measured using the Seahorse XF Cell Mito Stress Test Kit and the Seahorse XF^e^96 device (Seahorse Bioscience, North Billerica, MA, United States) according to the manufacturer’s protocol.

To test the effect of free fatty acids (FFAs) on the mitochondrial respiration in patient (*TMEM126B*, *NDUFS7*, *NDUFV2*, *NDUFA12*, *NDUFAF5*) and control (C1) fibroblasts, we seeded 12,000 fibroblasts per well, using eight replicate wells per condition, in a Seahorse XF 96-well plate. Cells attached overnight in normal DMEM (25 mM) growth media, supplemented with 10% FBS. Prior to assay, fibroblasts were incubated for 16 h in DMEM with glucose (25 mM), but with limited FBS (1%), where either 500 μM BSA-conjugated palmitic acid or oleic acid was added to the media of the treated conditions and unbound BSA was added to the untreated conditions. FFAs need to be BSA-conjugated (ratio FFA: BSA = 6:1) in order to be taken up by the cells. One hour before assay, media was replaced with Seahorse assay media containing 10 mM glucose, 2 mM glutamine, 1 mM pyruvate, 1% FBS, and OCR were measured using the Mito Stress Kit according to the manufacturer’s manual. Administration of oligomycin (2.0 μM) blocked complex V activity, and was used as a measure for ATP production (start resp. – oligomycin resp.). Uncoupling agent FCCP (1.0 μM) stimulated cells to their maximal respiration level and was used together with antimycin A/rotenone (0.5 μM), blocking complete OXPHOS respiration via complex I and III, as a measure for maximal respiratory capacity (FCCP resp. – antimycin A/rotenone resp.). Furthermore, OXPHOS spare capacity (FCCP resp. – start resp.) and proton leak (oligmycin resp. – antimycin A/rotenone resp.) were determined. OCR was measured at four time points after compound administration, where the 4th measurement was used to calculate statistical significance (*P* < 0.05). To correct for differences in proliferation rate, OCR’s were normalized to control according to protein quantity.

### Statistical Analysis

Statistical significance was calculated using a two-sample *t*-test, one-tailed, equal variance. A 95% confidence interval and alpha-level 0.05 was applied.

## Results

### Patient Clinic and OXPHOS Complex I Activity

A female from a non-consanguineous family had a motor development delay from birth onward, while mental milestones were reached in time. Muscle weakness and cramps in the legs developed and she had difficulties with walking. The muscle weakness could disappear after resting for 2 h. Exercise intolerance persisted, and at 14 years of age metabolic investigations indicated elevated levels of lactic acid (2.8–5 mmol/l). Alanine and pyruvic acid levels were normal. Muscle histochemistry showed an increased intensity and linear staining of SDH and COX in the subsarcolemmal regions. Furthermore, ragged red fibers were seen in approximately 40% of the muscle tissue. Electron microscopy indicated elongated, enlarged and abnormally shaped mitochondria (subsarcolemmal) and dispersed dense bodies with abnormal cristae and crystalline inclusions (Supplementary Figure S1). Complex I activity of the patient was approximately 20% of control muscle (**Table 1**) and increased activities were measured for CIV and CV. For the last 10 years the patient remained easily fatigued.

**Table 1:** Oxidative phosphorylation (OXPHOS) complex activities in patient muscle biopsy.

OXPHOS complex	Activity	Control SEM	% as to control SEM
I	0.76 (μmol NADH/min/g)	3.66 ± 0.22	21% (↓)
II	n.d. (μmol INT^+^/min/g)	723 ± 40	n.d.
II+III	7.43 (μmol cyt c/min/g)	5.20 ± 0.32	143% (↑)
IV	195.0 (k/min/g)	94.6 ± 3.5	206% (↑)
V	31.20 (μmol Pi/min/g)	11.90 ± 0.83	262% (↑)

### Dietary Studies

Bicycle endurance testing (15% of *W*_max_) on the patient’s (TMEM126B patient) usual diet indicated that muscle endurance was far below normal levels, where the test persisted 60 min with a mean oxygen consumption (VO_2mean_) of 7,45 ml/kg/min. Also the maximal exertion (*W*_max_: 60 W) and maximal oxygen consumption levels (VO_2max_: 13,0 ml/kg/min) were below normal level and Magnetic Resonance Phosphor Imaging (31P MRS) of the muscle showed delayed recovery of the phosphocreatine levels after exercise. Dietary intervention studies were performed to examine the effect of a high-carbohydrate diet (25% of energy from fat) vs. a high fat-diet (55% of energy from fat). During the 3 weeks of carbohydrate diet, the patient felt increasingly exhausted and activities of daily living were more difficult to perform than with her usual diet. Exercise testing showed that the patient’s bicycle endurance was 53% prolonged after the high-fat diet, compared to the carbohydrate diet (95 min vs. 62min), where the mean oxygen consumption was higher after the high-fat diet (VO_2mean_: 8,51 vs. 6,82 ml/kg/min). No differences were measured with the maximal exertion test (*W*_max_), although oxygen consumption was higher after the high-fat diet. Differences in endurance measured after direct substrate infusion showed that the patient sustained the bicycle test 38% longer (90 min vs. 65 min) on intra-lipid infusion compared to glucose infusion. The mean oxygen consumption (VO_2mean_) was higher with lipid infusion than glucose infusion (7,45 ml/kg/min vs. 5,09 ml/kg/min). Also in muscle strength, which was measured at different body parts, lipid-infusion seemed to have an advantage in terms of strength of the lower extremities, with less decline between subsequent measurements. More subjective, the patient stated that she felt physically better when consuming a high-fat diet (Supplementary Table S1).

### Whole-Exome Sequencing (WES)

Whole-exome sequencing analysis revealed a homozygous c.635G > T missense mutation in the *TMEM126B* gene, a known complex I assembly factor (NM_018480.4:c.635G > T, chr: 11, g.85347215). The resulting p.G212V amino-acid substitution is located in a transmembrane region at a position that is highly conserved in mammals (Nextprot: NX_Q8IUX1). In vertebrates, the conservation of Glycine, Serine, and Alanine at p.212 (belonging to a class of small size amino acids) suggested structural constraints on the size of the amino acid, which is violated by substitution with a larger valine residue. *In silico* pathogenicity predictions assigned highly damaging properties to the mutation at protein level (SIFT: 0.002, PROVEAN: -6.2, PF: 0.99). Mutation segregation analysis confirmed a recessive inheritance pattern as the mother was heterozygous carrier (Supplementary Figure S2). No DNA was available from the father and healthy brother, therefore the possibility of a hemizygous mutation was excluded by *TMEM126B* copy-number analysis (Supplementary Figure S3). The p.G212V mutation has an allele frequency of 0.1–0.2% (dbSNP: rs141542003) in dbSNP and ExAC databases.

### Complex I Assembly and Lentiviral Complementation

**FIGURE 1:**
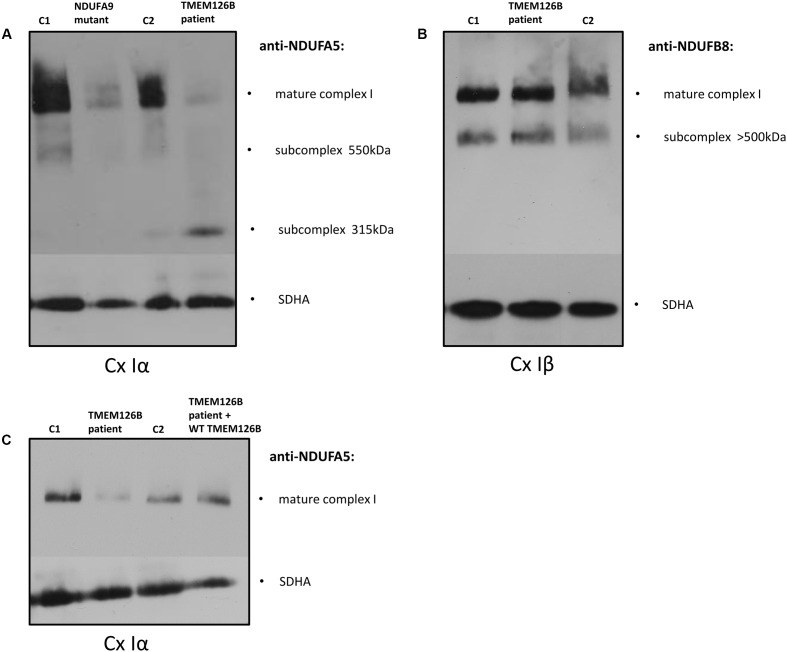
Blue-Native PAGE on fibroblasts of the patient (TMEM126B patient), 2 controls [control 1 (C1), control 2 (C2)] and NDUFA9 mutant. **(A)** Anti-bodies against NDUFA5 were used to detect mature complex I and assembly intermediates containing the Q module of the peripheral arm of complex I (Cx Iα). A loss of mature complex I and accumulation of the 315 kDa intermediate in the patient indicated impaired complex I assembly. **(B)** NDUFB8 antibodies detected assembly stages of the membrane arm of complex I (Cx Iβ), which showed normal NDUFB8 quantities in mature complex I, suggesting that assembly of the membrane arm (P-module) succeeded despite missing parts of the peripheral matrix arm [**(A,B)** are the representatives of two replicates]. **(C)** Patient fibroblasts (TMEM126B patient) were lentiviral transduced with wild-type TMEM126B (TMEM126B patient + WT TMEM126B) and showed a recovery of the amount of mature complex 1 when compared to healthy control fibroblast (C1 + C2) [**(C)** consists of a single experiment].

The effect of the *TMEM126B* mutation on complex I integrity was studied by Blue-Native PAGE electrophoresis. Mitochondrial fractions were isolated from fibroblasts of the patient (TMEM126B patient), two controls [control 1 (C1) and control 2 (C2)] and an *NDUFA9* mutant as a negative control. Antibodies against the subcomplex Iα (Cx Iα) subunit NDUFA5 were used to detect mature complex I and assembly intermediates containing this peripheral arm module (Q module) (Lazarou et al., 2009; Andrews et al., 2013; Rak and Rustin, 2014). A drastic loss of mature complex I and accumulation of the 315 kDa intermediate was observed, indicating impaired complex I assembly in the patient (**Figure 1A**). Antibodies against the subcomplex Iβ (Cx Iβ) subunit NDUFB8 were used to detect assembly stages of the membrane arm of complex I (P module) (Lazarou et al., 2009; Mimaki et al., 2012), and showed normal quantities of NDUFB8 in the mature complex I (**Figure 1B**). Complex II (SDHA) was used as a protein loading control. To provide evidence that defects in *TMEM126B* cause incomplete assembly of the peripheral arm of complex I, patient fibroblasts were rescued by lentiviral transduction with wild-type *TMEM126B* (isoform A, NM_018480.4). Blue-Native PAGE and immunoblotting with anti-NDUFA5 indicated that the amount of fully assembled complex I in patient fibroblasts was largely restored upon complementation (**Figure 1C**).

### Mitochondrial Respiration and Fatty-Acid Treatment

**FIGURE 2:**
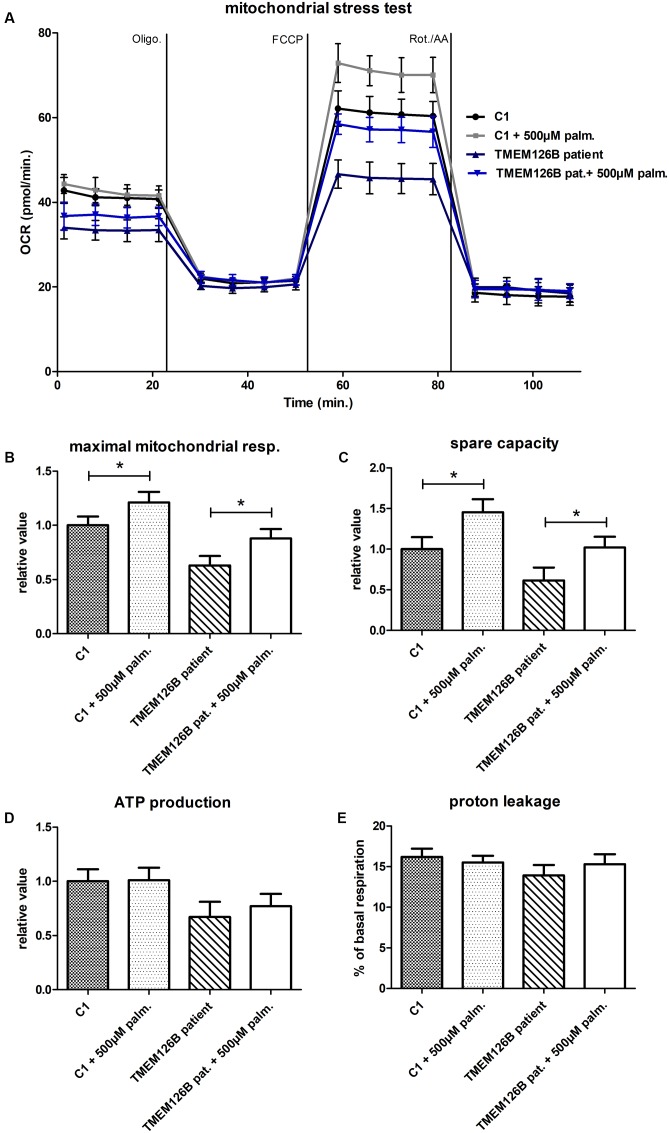
Mitochondrial stress test in TMEM126B patient fibroblasts. **(A)** Oxygen consumption rates (pMoles/min) measured in patient and healthy control fibroblasts (C1) after 16 h treatment with 500 μM palmitic acid. **(B)** Maximal respiratory capacity in both control and patient fibroblasts increased with 21 and 25%, respectively, upon treatment with palmitic acid, where respiration in the TMEM126B patient restored from 63 to 88% of the control level. **(C)** The measured increase in maximal respiration resulted in a 45 and 67% increase in respiratory spare capacity in control and patient cells, respectively. **(D)** ATP production in patient fibroblasts was 63% of the untreated control level and was not significantly altered by palmitic acid treatment. **(E)** Also proton leakage was not significantly changed, where basal respiration due to non-coupled ATP production was never higher than 17%. ^∗^Statistically significant.

A mitochondrial stress test was performed to examine OXPHOS capacity in patient fibroblasts. OCR measurements indicated decreased ATP production and decreased maximum respiratory capacity in patient fibroblast compared to control lines (C1 and C2) (Supplementary Figure S4A). As indicated by the extracellular acidification rates (ECAR), patient fibroblasts were more dependent on glycolytic metabolism than control fibroblasts (Supplementary Figure S4B). Respiratory findings therefore corresponded with the observed complex I assembly defect in the patient’s fibroblasts. To test if unsaturated or saturated fatty acids have an effect on OXPHOS capacity, patient fibroblasts were treated with 500 μM oleic acid or palmitic acid, respectively, for 16 h prior to measuring the oxygen consumption under different mitochondrial stress conditions. Whereas oleic acid did not have any significant effect on the OCR in patient or control cells (C1) (Supplementary Figure S5), pre-incubation with palmitic acid resulted in a significant increase of 25% in maximal respiratory capacity in *TMEM126B* patient fibroblasts, from 63 to 88% of the untreated control level (**Figures 2A,B**). In control fibroblasts, palmitic acid treatment significantly increased maximal respiratory capacity with 21%. This increase in maximal respiration was accompanied by an increase in respiratory spare capacity of 45 and 67% in control and patient cells, respectively (**Figure 2C**). ATP production in patient fibroblasts was 63% of the untreated control condition and increased 10% as a result of palmitic acid treatment, however, this was not statistically significant (**Figure 2D**). Proton leakage was not significantly changed, where basal respiration due to non-coupled ATP production was never higher than 17% (**Figure 2E**).

**FIGURE 3:**
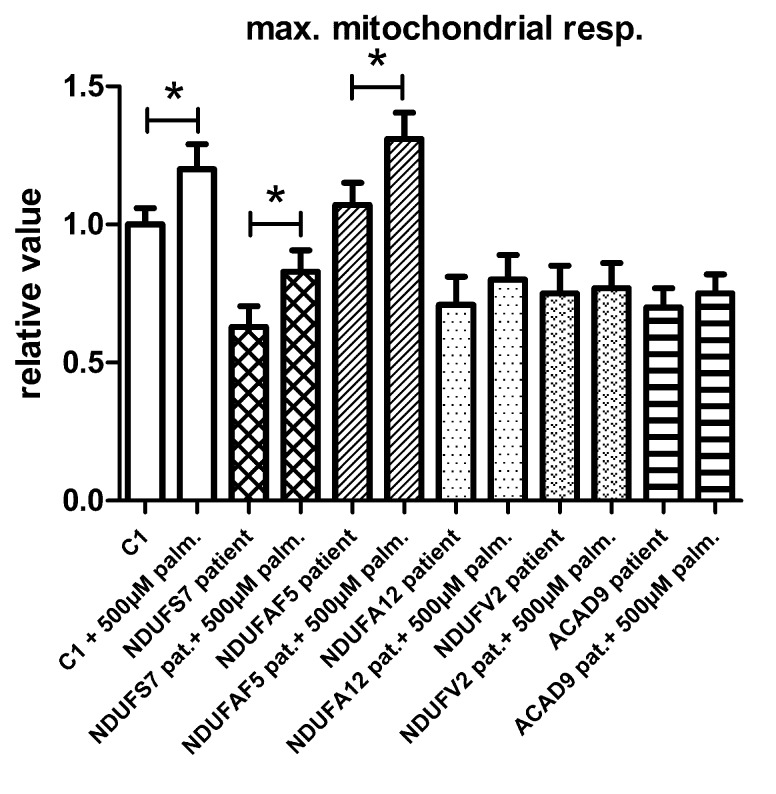
The effect of palmitic acid on the maximal respiratory capacity in fibroblasts. Control (C1) and patient fibroblasts with defects in the complex I subunit and assembly factor genes NDUFS7 (p.V122M), NDUFAF5 (p.L159F), NDUFA12 (c.83dup, frameshift), NDUFV2 (p.A183T), and ACAD9 (p.R532W) were treated with palmitic acid prior to seahorse analysis. NDUFS7 and NDUFAF5 cells were responsive to palmitic acid as a significant increase of, respectively, 32 and 22% in maximal respiratory capacity was measured. No significant changes in maximal respiration were measured in NDUFA12, NDUFV2, and ACAD9 defective fibroblasts. ^∗^Statistically significant.

To test if the increase in maximal respiratory capacity after palmitic acid treatment in control and *TMEM126B* patient fibroblasts (p.G212V) was related to complex I function in general, we performed similar respiratory measurements in patient fibroblasts with a variety of complex I gene defects, all patients being CI deficient in muscle biopsy, but not all of them with a significant respiratory deficiency in fibroblasts (**Figure 3**). This included patients with mutations in the subunits *NDUFS7* (p.V122M), *NDUFA12* (c.83dup, frameshift), *NDUFV2* (p.A183T) and assembly factors *ACAD9* (p.R532W) and *NDUFAF5* (p.L159F). As indicated in **Figure 3** and Supplementary Figure S6, fibroblast with pathogenic mutations in *NDUFS7* and *NDUFAF5* showed a significant increase of 32% (from 63 to 83% of untreated control) and 22% (from 107 to 131% of untreated control), respectively, in maximal respiratory capacity upon palmitic acid treatment. No significant influence of palmitic acid on maximal respiration was measured in the *NDUFA12* (71% of control), *NDUFV2* (75% of control), and *ACAD9* (70% of control) fibroblasts.

## Discussion

**FIGURE 4:**
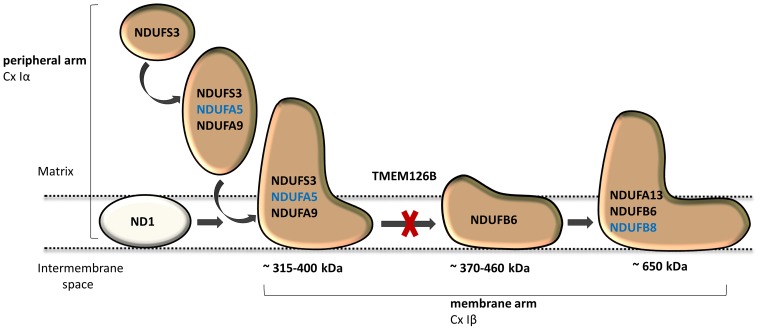
TMEM126B functions as an early complex I assembly factor. TMEM126B is required for the joining of the ∼315 and ∼370 kDa assembly intermediates (previously estimated ∼400 and ∼460 kDa, respectively). BN-Page on TMEM126B defective patient fibroblasts indicated accumulation of NDUFA5 (Q-module) in the ∼315 kDa intermediate and a drastic decrease in complex I, indicating that part of the peripheral arm was missing. Membrane arm subunit NDUFB8 (N-module) was present in normal quantities in mature complex I, indicating that assembly of the complex I membrane arm succeeded despite missing parts of the peripheral arm.

Dietary studies in a complex I deficient patient with exercise intolerance indicated beneficial effects of a high-fat diet on the patient’s muscle endurance. WES analysis was performed to characterize the genetic defect and identified a highly damaging, homozygous p.G212V substitution in the transmembrane region of TMEM126B (NM_018480.4:c.635G > T). Our data show that the p.G212V mutation in *TMEM126B* causes an accumulation of the 315 kDa complex, containing the peripheral arm subunit NDUFA5 (Cx Iα), possibly via interrupted merging of the 315 and 370 kDa subcomplexes (**Figure 4**). This resulted in a drastic loss of mature complex I in patient fibroblasts. The resulting complex I deficiency could be rescued by wild-type *TMEM126B*, confirming causality. Last year, the first patients with *TMEM126B* defects were identified, all carrying the p.G212V mutation in hetero- or homozygous fashion (Alston et al., 2016; Sanchez-Caballero et al., 2016), possibly due to the relatively high allele frequency in the European-American population (0.1–0.2%). Our data show that biochemical and clinical differences exist among the homozygous p.G212V patients (**Table 2**). In line with the complex I deficiency described for subject 6 of the previously reported cases (Alston et al., 2016), we observed a respiratory deficiency in the patient’s fibroblasts. In contrast, the second case, subject 1, showed a muscle specific respiratory deficiency. Clinical symptoms in our patient were similar to subject 1, as both manifested with mild exercise intolerance (**Table 2**). In contrast, the other reported case suffered from more severe, heterogeneous symptoms, involving cardiomyopathy, renal failure and growth deficits. Yet, it is most likely that the p.G212V substitution explains a relatively mild phenotype, as all other reported *TMEM126B* patients were described to suffer mainly from exercise intolerance, even in combination with more harmful nonsense mutations. Most likely, in our opinion, the *TMEM126B* mutation by itself cannot explain the extended phenotype of the severely affected patient.

**Table 2:** Clinical and biochemical characteristics of the homozygous p.G212V TMEM126B patients.

Patient	Mutation	Clinic	Muscle morphology	Muscle CI activity	Fibroblast OXPHOS	CI assembly
Index patient Non-consanguineous	Homozygous pG212V	Exercise intolerance (mild)	Subsarcolemmal accumulation mitochondria, RRF	CI↓ (C1: 21%, as to control SEM)	OXHOS capacity (OCR)↓	Incomplete assembly of CI, part of peripheral arm missing
Subject 1 Non-consanguineous (Alston et al., 2016)	Homozygous pG212V	Exercise intolerance (mild)	Subsarcolemmal accumulation mitochondria, RRF	CI↓ (C1: 36%, as to control SEM)	CI activity (normal)	Fibroblasts: no assembly defect; muscle: complete lack of mature CI or membrane arm module
Subject 6 Non-consanguineous (Alston et al., 2016)	Homozygous pG212V	Gastroesophageal reflux, renal failure, cardiomyopathy, failure to thrive, growth deficits	Subsarcolemmal accumulation mitochondria, RRF	CI↓ (C1: 17%, as to control SEM)	CI activity↓	n.d.

Considerable differences in complex I assembly were observed in *TMEM126B* patients (**Table 3**). Where our data indicated that an incomplete constitution of complex I affected mitochondrial respiration in both muscle and fibroblasts, a previously reported homozygous p.G212V patient did not show an assembly defect in fibroblasts at all. Subunits from both the early ∼315 kDa peripheral arm intermediate (NDUFA9, NDUFS3; Q-module) and the ∼370 kDa membrane arm (NDUFB6; P-module) were present in mature complex I in normal quantities. In contrast, complex I in muscle tissue showed an almost complete lack of the membrane arm subunit NDUFB8 (∼650 kDa intermediate), likely indicating a decrease in the total amount of mature complex I (Tabel 3 and **Figure 4**). The p.G212V mutation in combination with more harmful nonsense and splice mutations was shown to cause a decrease in total amount of mature complex I, as subunits of the peripheral arm (NDUFS3, NDUFA9) and membrane arm were significantly decreased (NDUFB8, NDUFA13, and NDUFB6) (McKenzie and Ryan, 2010; Mimaki et al., 2012). This is striking as the severity of the assembly defect is apparently not defined by the mildest mutation. As shown in **Figure 4**, the current complex I assembly model is suggested to start with the joining of a ∼315 kDa subcomplex, containing the peripheral arm subunit NDUFA5 (Q-module, Cx Iα), and a ∼370 kDa subcomplex, containing the membrane arm subunit NDUFB8 (N-module, Cx Iβ) (previously estimated ∼400 and ∼460 kDa, respectively) (Lazarou et al., 2009; Mimaki et al., 2012; Andrews et al., 2013; Rhein et al., 2016). The MCIA complex, containing TMEM126B, was found to be associated with the ∼370 kDa assembly intermediate. siRNA-knockdown experiments in human 143B cells confirmed a role for TMEM126B as an early extrinsic assembly factor as a significant loss of the peripheral arm subunit NDUFA5 in complex I occurred together with an accumulation of the ∼315 kDa subcomplex (Andrews et al., 2013). Membrane arm subunit NDUFB8 revealed elevated levels of the ∼370 kDa subcomplex and decreased amounts of the larger ∼550 kDa intermediate, suggesting that TMEM126B is required for the joining of the ∼315 and ∼370 kDa subcomplexes during early complex I assembly. Yet, as NDUFB8 was still abundant in mature complex I, it is likely that part of the membrane arm assembly succeeded.

**Table 3:** The effect of homozygous and compound heterozygous p.G212V TMEM126B mutations on complex I assembly.

Homozygous patients	Mutation	Peripheral arm subunits present in early 315–400 kDa intermediates (fibroblasts)	Membrane arm subunits present in >370–460 kDa intermediates (fibroblasts)	Muscle BN-PAGE
**Index patient**	pG212V	NDUFA5↓	NDUFB8 (normal)	n.d.
Subject 1 (Alston et al., 2016)	pG212V	NDUFA9 (normal), NDUFS3 (normal)	NDUFB6 (normal)	NDUFB8↓ (∼370 kDa membrane arm intermediate)
**Heterozygous patients**				
Subject 1 + 3 (Sanchez-Caballero et al., 2016)	pG212V; pAsp133Asn (splice variant)	NDUFS3↓ + *accumulation small subcomplexes*	NDUFB8↓, NDUFA13↓	n.d.
Subject 2 (Sanchez-Caballero et al., 2016)	pG212V; pGln70^∗^(nonsense)	n.d.	NDUFB8↓, NDUFA13↓	n.d.
Subject 2 + 3 (Alston et al., 2016)	pG212V; pAsn134Ilefs^∗^2 (nonsense)	NDUFA9↓, NDUFS3↓	NDUFB6↓	n.d.

Our patient showed improved muscle endurance and strength on a high-fat diet compared to a high carbohydrate diet. For the clinical benefits of high-fat intake in complex I deficiency anecdotic reports exist and have led to the clinical use of different therapeutic high-fat diets and ketogenic diets (low in carbohydrates). Not all complex I deficient patients seem to benefit from such diet, but it is currently unclear which complex I gene defects respond to these therapies and how mitochondrial function is influenced by an increase in fatty-acids or ketone bodies. FFAs are an important source for mitochondrial energy production, but it has been difficult to elucidate their effect on mitochondrial function as contradictory effects have been reported in terms of mitochondrial oxygen consumption and toxicity (Hirabara et al., 2006; Maassen et al., 2007; Abe et al., 2013). In line with mitochondrial respiration data from mouse podocytes, mouse neonatal myocytes and Normal Human Dermal Fibroblasts (NHDF), we measured a significant increase in oxygen consumption of healthy control fibroblasts (NHDF) due to palmitic acid treatment (Zhang et al., 2012; Abe et al., 2013; Pfleger et al., 2015). Maximal mitochondrial respiration capacity and spare capacity significantly increased with palmitic acid. Besides the maximal respiratory capacity, the spare mitochondrial respiratory capacity (or reserve capacity) has been reported as an important part of mitochondrial function, especially when cells are subject to stress, demanding increased levels of ATP to maintain cellular functions. Improvements in spare respiratory capacity can lead to increased ATP production to overcome stress and enhance cell viability, where complex II was suggested to be an important source of this reserve capacity (Pfleger et al., 2015; Yamamoto et al., 2016). As there have been indications that long-chain fatty acid can increase proton leak in liver mitochondria, possibly via mitochondrial uncoupling by the ATP/ADP and aspartate/glutamate antiporters, we have assessed whether this mito-toxic effect also occurred during our respiratory assay. We measured no significant increase in proton leakage due to palmitic acid treatment in the fibroblasts, which could otherwise have influenced the measured maximal respiratory levels (Samartsev et al., 1997; Hirabara et al., 2006).

Our data show that, despite the severe complex I assembly defect, maximal mitochondrial respiration and spare capacity in TMEM126B defective patient cells significantly improved due to palmitic acid treatment (up to 88% of untreated control level), therewith supporting the dietary intervention studies in this patient. Interestingly, the unsaturated fatty acid oleic acid did not have any effect on mitochondrial respiration. The same assay was used for testing patient fibroblasts with different complex I defects, affecting both subunits and assembly factors, and showed that only part of the complex I defects are responsive to palmitic acid treatment, including NDUFS7 and NDUFAF5 defects. We demonstrate that this is an easy assay, which could be used as a personal medicine tool in the clinic to discriminate complex I deficient patients who might benefit from the intake of palmitic-acid, and those who could not (Golubitzky et al., 2011). In line with the current knowledge on FFAs, we show that the effects strongly differ among the individual FFAs, as the unsaturated FFA oleic acid did not have any effect on mitochondrial respiration at all. Whereas in clinical practice, different diets are subsequently tested in the patient, without knowing the effective components, our cell-line assay could overcome these limitations as a tool to identify responsiveness to specific FFAs (or other nutrients or drugs), thereby providing a personalized treatment approach. Although, therapeutic high-fat diets could be a possible way to increase the intake of palmitic acid in responsive patients, one should be aware that individual FFAs could behave differently as part of a complete diet (Watt et al., 2012).

It is difficult to fully explain mechanistically the differences in reaction by the data available. A proposed mechanism for FFAs on mitochondrial function involved the induction of the PPARγ pathway. The saturated FFAs deconoic acid and palmitic acid, but not the unsaturated FFA oleic acid, were previously shown to increase PPARγ protein expression or, as an agonist, stimulate its activity. PPARγ can stimulate mitochondrial biogenesis and proliferation, and could therefore increase cellular respiratory capacity (Miglio et al., 2009; Hughes et al., 2014; Kanabus et al., 2016). However, this would be a general effect, suggesting that this mechanism cannot explain the differences between the patients, because it would imply that all CI deficient patients in our assay should have reacted to the treatment, independent of the assembly defect. An alternative explanation could be an increase in FFA beta-oxidation, which results in relatively higher FADH_2_:NADH ratio’s (1:1) than glycolysis (1:3). As FADH_2_ can directly donate its electrons to complex II of the OXPHOS system, it could bypass the complex I defect, therewith improving mitochondrial respiration (Roef et al., 2002; Paoli et al., 2014; Steriade et al., 2014). Although the latter mechanism might contribute to an increase in respiratory capacity, this cannot explain our observation that only part of the complex I defects responds to palmitic acid treatment (**Table 4**). Complex I specific effects have previously been reported for both FFA’s and ketone bodies, where decanoic acid was shown to increase complex I activity in healthy neuronal cells (Hughes et al., 2014), and ketone bodies were shown to alleviate mitochondrial dysfunction by increasing complex I activity in MELAS cells (neuronal cybrid cells with m.3243A > G) via restoration of the complex I assembly defect (Frey et al., 2017). Interestingly, we found genetic defects in assembly factors and subunit (NDUFAF5, NDUFS7, and TMEM126B) involved in early assembly of the peripheral arm of complex I responsive to palmitic acid (**Table 4**) (Mimaki et al., 2012; Rhein et al., 2016). Although ACAD9, which is functional in the same MCIA complex as TMEM126B, did not respond to the treatment, this protein is known to have another crucial function as a catalyzer in the fatty acid beta-oxidation (McAndrew et al., 2008), making its role more complicated. The non-responsive gene defects affecting the subunits NDUFA12 and NDUFV2 are incorporated at a later assembly stage (>800 kDa) (Mimaki et al., 2012), suggesting a compensating role for FFA in the early assembly process. However, current data is still too limited to draw a definite conclusion on the mechanism and testing more complex I defects will be needed.

**Table 4:** The effect of palmitic acid on respiratory capacity in patient fibroblasts with different CI defects.

Complex I defect	Patient clinic	M/F complex I activity	OXPHOS deficiency on seahorse (F)	Significant effect of palmitic acid on max. resp.
Healthy control (NHDF)	n.a.	Normal	No	Yes (20–21% increase)
TMEM126B: p.G212V (c.635G > T) substitution	Exercise intolerance	M: CI = 21%	Yes	Yes (25% increase)
NDUFS7: p.V122M (c.364G > A) substitution	Leigh	M: CI = 37% (mild decrease CIII)	Yes	Yes (32% increase)
NDUFAF5: p.Leu159Phe (c.477A > C) substitution	Leigh	M: CI = 32%	No	Yes (22% increase)
NDUFA12: homozygous c.83dup, frameshift	Leigh	M: CI = 16%, F: CI = 47%	Yes	No
ACAD9: p.Arg532Tryp (c.1594C > T) substitution	Exercise intolerance, fatigue	M: CI = 30%, F: CI = 50%	Yes	No
NDUFV2: p.Ala183Thr (c.547G > A) substitution	White matter degeneration	M: CI = 47%	Yes	No

*“Blue” colored row = assembly factor defect, “gray” colored row = subunit defect, M = muscle, F = fibroblast*.

Our data shows at a biochemical level that palmitic acid intake, possibly also as part of a therapeutic high fat diet, can be beneficial for patients with a defect in assembly factor TMEM126B and that our cell line assay will be a useful approach for the selection of patients, who might potentially benefit from this therapeutic diet. It is obvious that this cell line assay could also be extended to test responsiveness of patients to other nutrients and drugs, using mitochondrial respiration as outcome measure.

## Ethics Statement

The study was performed on the fibroblast cell lines with informed consent of the participants on the use of rest material of routine clinical procedures for research, as approved by the local ethical committee of Maastricht University Medical Centre. Written informed consent, in accordance with the Declaration of Helsinki, was obtained from the TMEM126B patient.

## Author Contributions

RS and DH were involved in analysis and interpretation of WES data. TT, MG, KS, and FvT performed biochemical read-out studies and complementation studies to characterize the genetic defect. RV was responsible for cellular morphological analysis. TT and RK performed cellular respiratory analysis and compound testing. HS, SS, EH, and IdC performed patient diet studies, patient contact, clinical data collection. HS and IdC were responsible for the research plan and intellectual content of the manuscript. All authors have critically revised the manuscript and approved the final version.

## Conflict of Interest Statement

The authors declare that the research was conducted in the absence of any commercial or financial relationships that could be construed as a potential conflict of interest.
