# Supplementary material for: Selection and Characterization of Palmitic Acid Responsive Patients with an OXPHOS Complex I Defect
Source: Front Mol Neurosci. 2017 Oct 18;10:336. doi: 10.3389/fnmol.2017.00336 (PMC5651253; doi:10.3389/fnmol.2017.00336)
Supplement: Supplementary file 1 [file Data_Sheet_1.PDF]

Supplementary data:

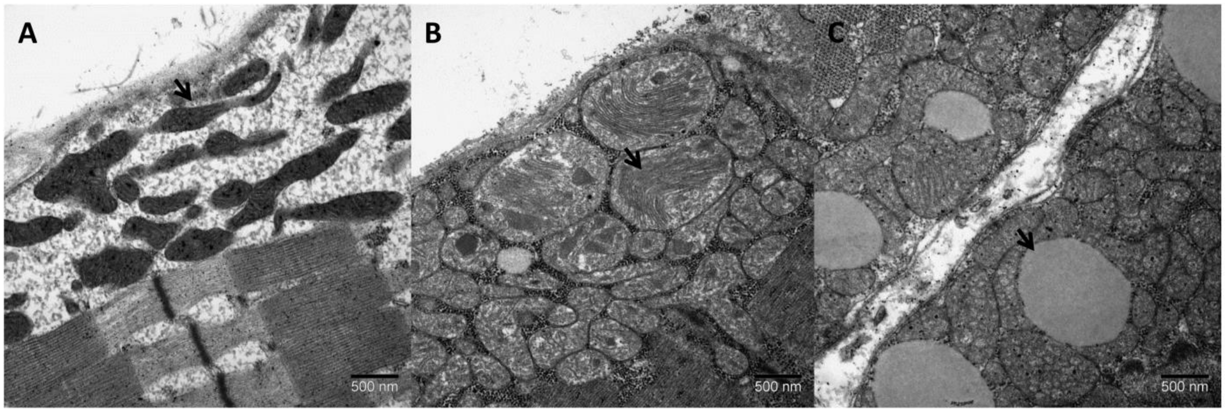

Figure S1: Electron microscopy on patient muscle biopsy (22,000x magnification) indicated (A) elongated and abnormally shaped mitochondria in the subsarcolemmal regions (arrow), (B) enlarged mitochondria and dispersed dense bodies with abnormal cristae and crystalline inclusions (arrow). (C) Large lipid droplets were present between the mitochondria (arrow).

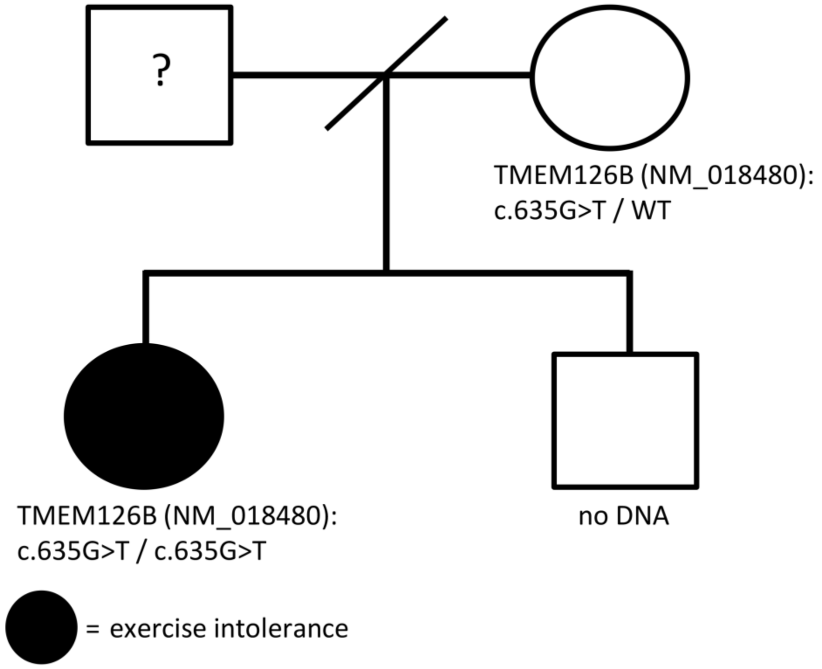

Figure S2: Family of the patient with exercise intolerance and complex I deficiency. All family members, except the patient, were unaffected. Sanger sequencing confirmed that the patient was homozygous for the p.G212V substitution in TMEM126B (NM_018480.4:c.635G>T). Her mother was heterozygous. Other family members were not available for analysis.

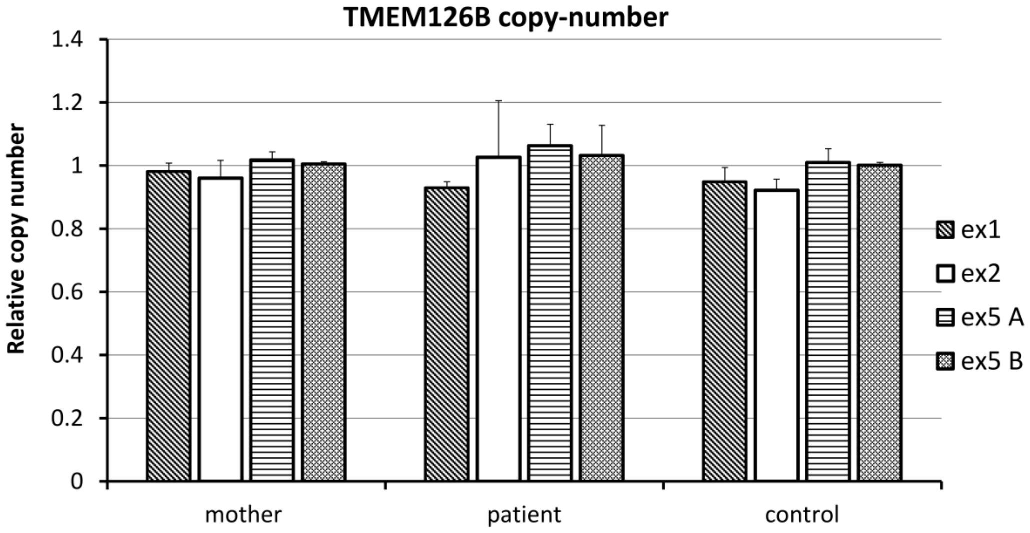

Figure S3: TMEM126B copy-number was quantified in the TMEM126B patient, mother of the patient, and control by the 7900HT Fast Real-Time PCR System, normalizing to B2M (beta-2-microglobulin gene, NC_000015.10). Primers were designed in TMEM126B exon 1, 2 and 5 (Supplementary Table S2) and copy numbers were relative to the heterozygous mother. No indications for hemizygosity were found.

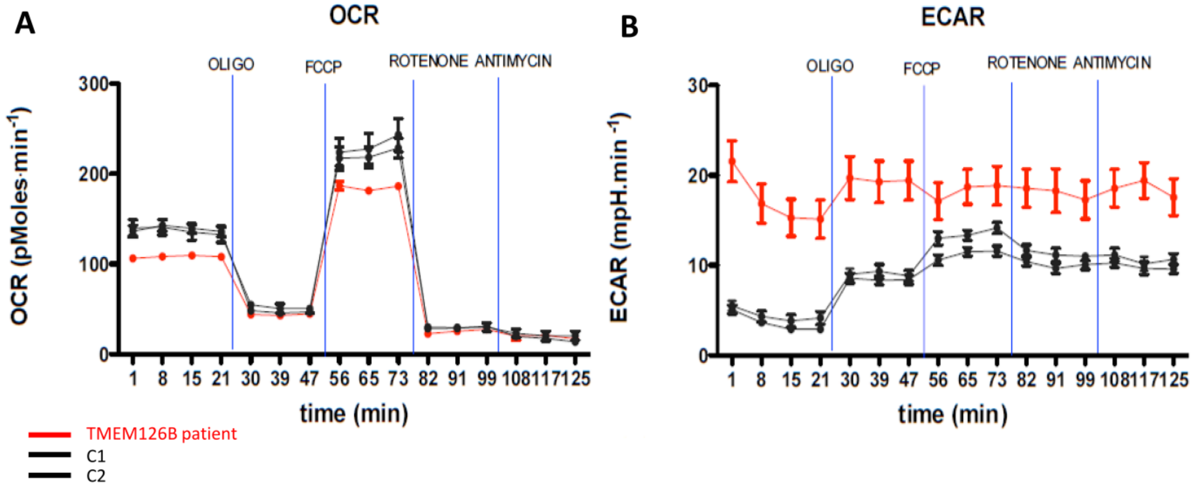

Figure S4: (A) Oxygen consumption rate (pMoles/min) and (B) acidification rates (mpH/min) measured in patient fibroblasts (TMEM126B patient) and healty control fibroblasts (C1 and C2) on the Seahorse system. The patient showed normal proton leakage, whereas basal respiration levels, ATP production and maximum respiratory capacity were decreased. Patient fibroblasts were more dependent on glycolytic metabolism than healthy controls.

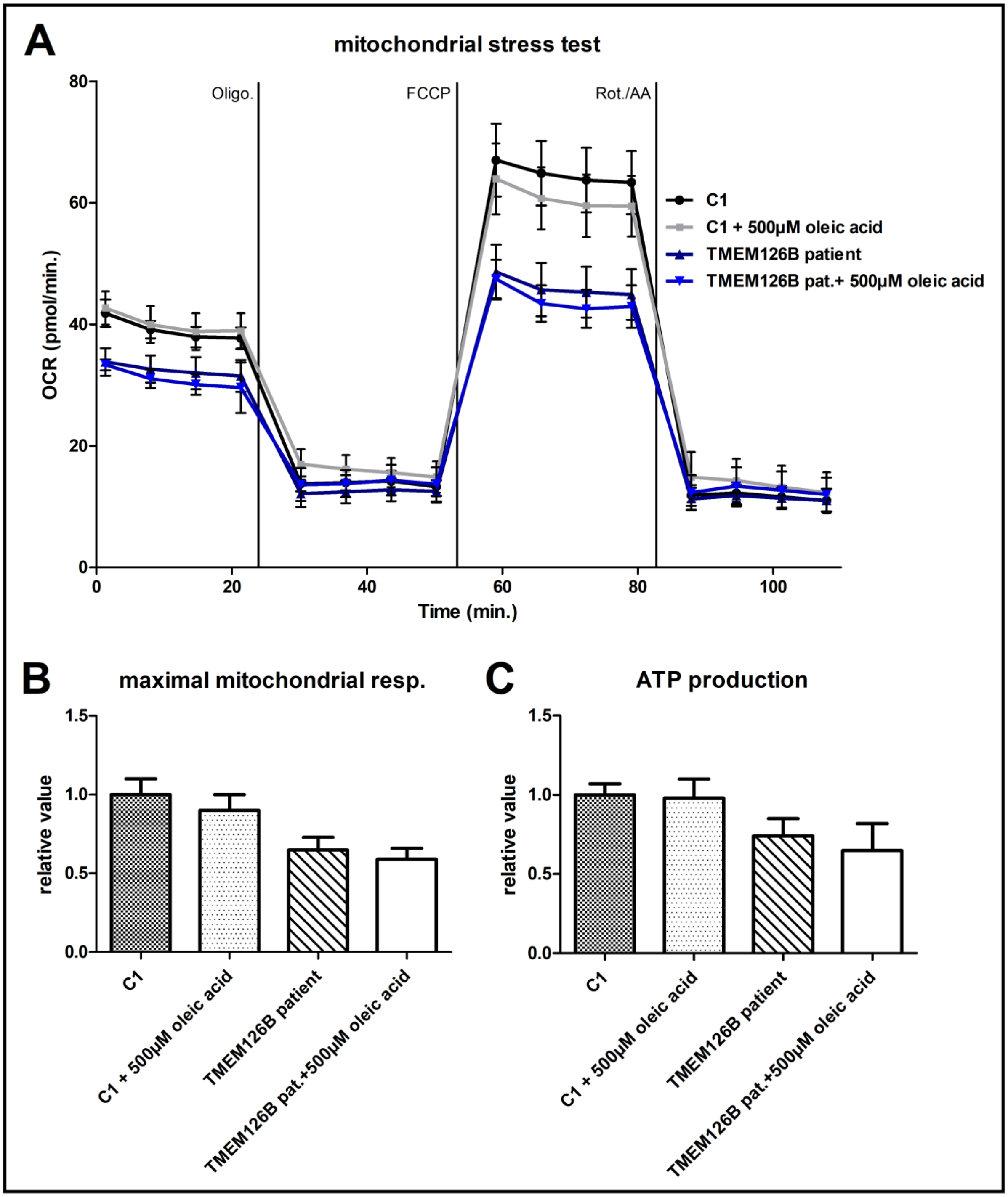

Figure S5: Oxygen consumption rates (pMoles/min) measured in TMEM126B patient fibroblasts and healthy control fibroblasts (C1) after 16h treatment with 500 µM oleic acid. No significant changes in OCR were observed as a result of oleic acid treatment.

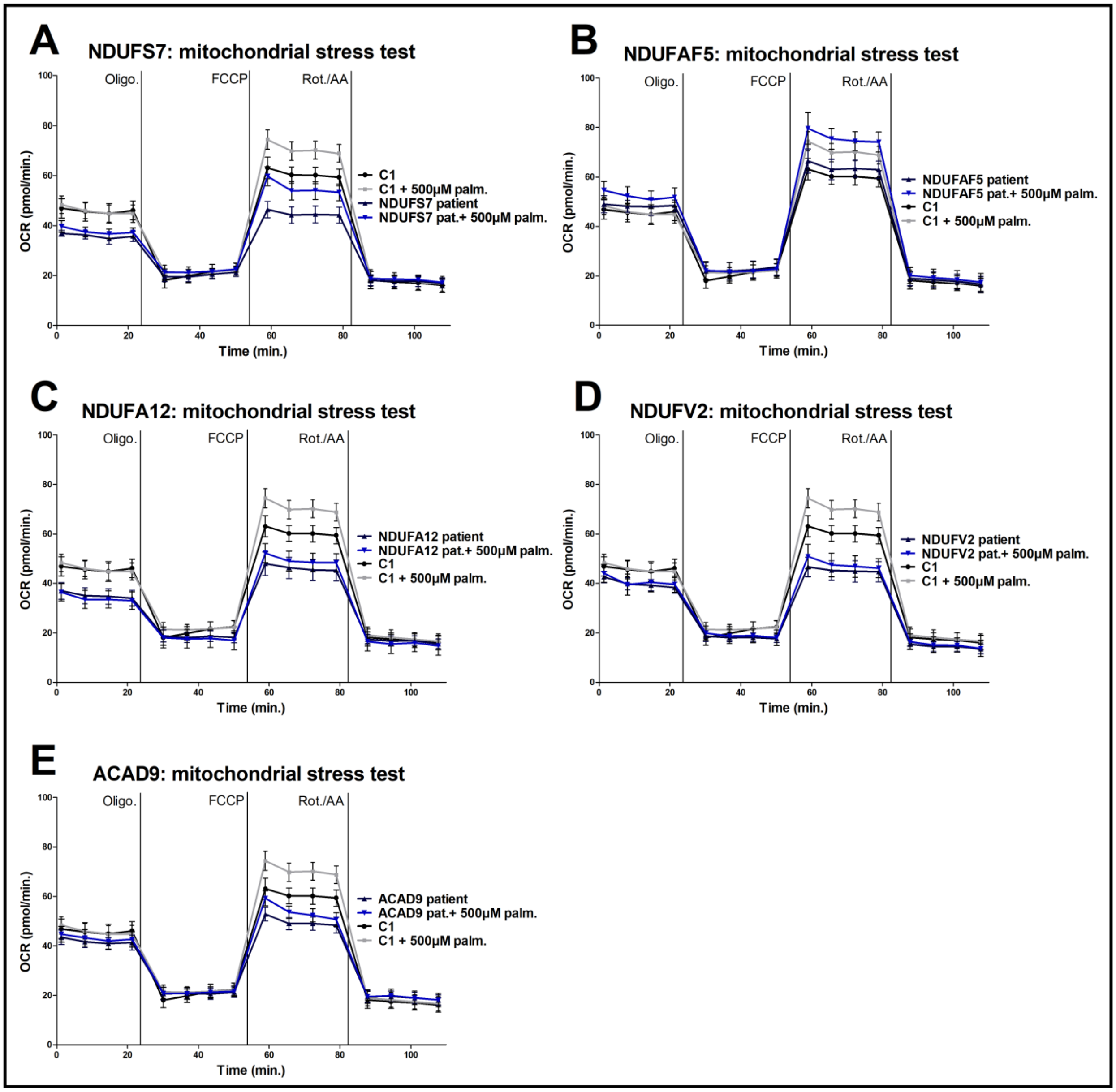

Figure S6: Oxygen consumption rates (pMoles/min) measured in healthy control fibroblasts (C1) and patient fibroblasts with different CI defects, all resulting in a CI deficiency in muscle. OCR was measured after 16h treatment with 500 µM palmitate. A significant increase in maximal respiratory capacity was measured in (A) NDUFS7 and (B) NDUFAF5 patient cells after palmitate treatment, whereas (C) NDUFA12, (D) NDUFV2, and (E) ACAD9 defective patient cells were not significantly influenced.

Table S1: Overview of the dietary study results.

**Diet**	**Persistence (min.) of bicycle endurance test at 15% of W_{max}**	**Mean oxygen consumption (VO_{2mean}) during bicycle endurance test (ml/kg/min.)**
usual diet (34,5% of energy from fat)	60min.	7,45 ml/kg/min.
3 weeks high-carbohydrate diet (25,0% of energy from fat)	62min.	6,82 ml/kg/min.
3 weeks high-fat diet (55,0% of energy from fat)	95min. (58%↑ and 53%↑ with respect to usual diet and high carbohydrate diet, respectively)	8,51 ml/kg/min. (14%↑ and 25%↑ with respect to usual diet and high carbohydrate diet, respectively)
**Substrate infusion**		
glucose infusion (10 mg/kg/min.)	65min.	5,09 ml/kg/min.
lipid infusion (3,7 mg/kg/min.)	90min. (38%↑ with respect to glucose infusion)	7,45 ml/kg/min. (46%↑ with respect to glucose infusion)<br>• higher muscle strength of the lower extremities

Table S2: Primers for Sanger sequencing.

**Sanger primer**	**5'-3' sequence (M13-tailed)**
FWD (ex5)	TGTAAAACGACGGCCAGTCCACCAAAAGGAAGGGTTTT
REV (ex5)	CAGGAAACAGCTATGACCCAGCAATGCCTGAGTCCTCT

Table S3: Primers for TMEM126B copy-number determination.

**primer**	**Fwd primer (5'-3')**	**Rev primer (5'-3')**
exon 1	TAAGCCAAGGGATTCAGGTG	CATGGACTTGGGAGCTGTTT
exon 2	CGACACAGTACCTGGCACAC	AGAGAAGGACTGGGCTGACC
exon 5 A	TGACGCTTTGTCAAACACAA	TCAAGTGTCTCTTCAAATACTGCAT
exon 5 B	AGCATTCATATCCAGGAGAGGT	CCAAACATAATCTGAAAGACTAGAGG
